# Pericoronary adipose tissue attenuation predicts compositional plaque changes: a 12-month longitudinal study in individuals with type 2 diabetes without symptoms or known coronary artery disease

**DOI:** 10.1186/s12933-025-02694-9

**Published:** 2025-03-28

**Authors:** Katrine Schultz Overgaard, Thomas Rueskov Andersen, Laurits Juhl Heinsen, Gokulan Pararajasingam, Roda Abdulkadir Mohamed, Freja Sønder Madsen, Irmelin Irene Aagaard Biesenbach, Kurt Højlund, Jess Lambrechtsen, Søren Auscher, Kenneth Egstrup

**Affiliations:** 1 Cardiovascular Research Unit, Odense University Hospital Svendborg, Baagøes Allé 15, 5700 Svendborg, Denmark; 2 Department of Clinical Research, Faculty of Health Sciences, University of Southern Denmark, Odense, Denmark; 3 Research unit of SDCO - Steno Diabetes Center Odense, Odense University Hospital, 5000 Odense C, Denmark

**Keywords:** Pericoronary adipose tissue, Inflammation, Type 2 diabetes mellitus, Coronary CT angiography, Coronary artery disease, Atherosclerosis progression, Plaque burden, Plaque vulnerability, Non-calcified plaque progression.

## Abstract

**Background:**

Pericoronary adipose tissue attenuation (PCATa), derived from coronary computed tomography angiography (CCTA), is a novel marker of inflammation in the coronary arteries. Patients with type 2 diabetes mellitus (T2DM) are at elevated risk of coronary artery disease (CAD), potentially due to systemic inflammation. This study evaluated whether baseline PCATa predicts changes in plaque composition and burden over 12 months.

**Methods:**

This prospective longitudinal study included 200 participants with T2DM, who had neither symptoms nor a prior diagnosis of CAD (mean age 61 ± 9.4 years, 72% male). PCATa was measured at the baseline scan along the proximal 40 mm of each major coronary artery, and the values were averaged to calculate the participant-level PCATa. High PCATa levels were determined using the validated cut-off of -70.1 Hounsfield units. Compositional plaque changes were quantified as the differences between baseline and 12-month scans, and plaque burden was calculated as the normalized atheroma volume. Multivariable regression analyses assessed the associations between baseline PCATa and compositional plaque changes and evaluated risk factors, including high PCATa, in predicting non-calcified plaque burden progression.

**Results:**

Plaque compositional volumes and burden increased over 12 months, while PCATa remained stable. After multivariable adjustments, baseline PCATa was significantly associated with changes in total plaque volume (β = 0.005, *p* = 0.005), non-calcified plaque volume (β = 0.006, *p* = 0.007), total plaque burden (β = 1.7, *p* = 0.007), and non-calcified plaque burden (β = 2.0, *p* = 0.006), but not with calcified plaque volume or burden. High baseline PCATa was observed in 44 participants (22%) and was the only independent predictor of non-calcified plaque burden progression (odds ratio 3.5, *p* = 0.002).

**Conclusions:**

Baseline PCATa is significantly associated with increases in total and non-calcified plaque volumes and burden over 12 months in participants with T2DM without symptoms or known CAD. High PCATa levels uniquely predict non-calcified plaque burden progression, suggesting that PCATa may serve as a marker for subclinical atherosclerosis progression. This warrants further investigation into PCATa for cardiovascular risk assessment, particularly in high-risk populations such as individuals with T2DM.

**Trial registration:**

Trial registration: NCT06644651.

**Graphical abstract:**

PCATa = Pericoronary Adipose Tissue attenuation. T2DM = Type 2 diabetes mellitus. CAD = Coronary Artery Disease. *N* = numbers. CCTA = Coronary CT angiography. Created in BioRender.

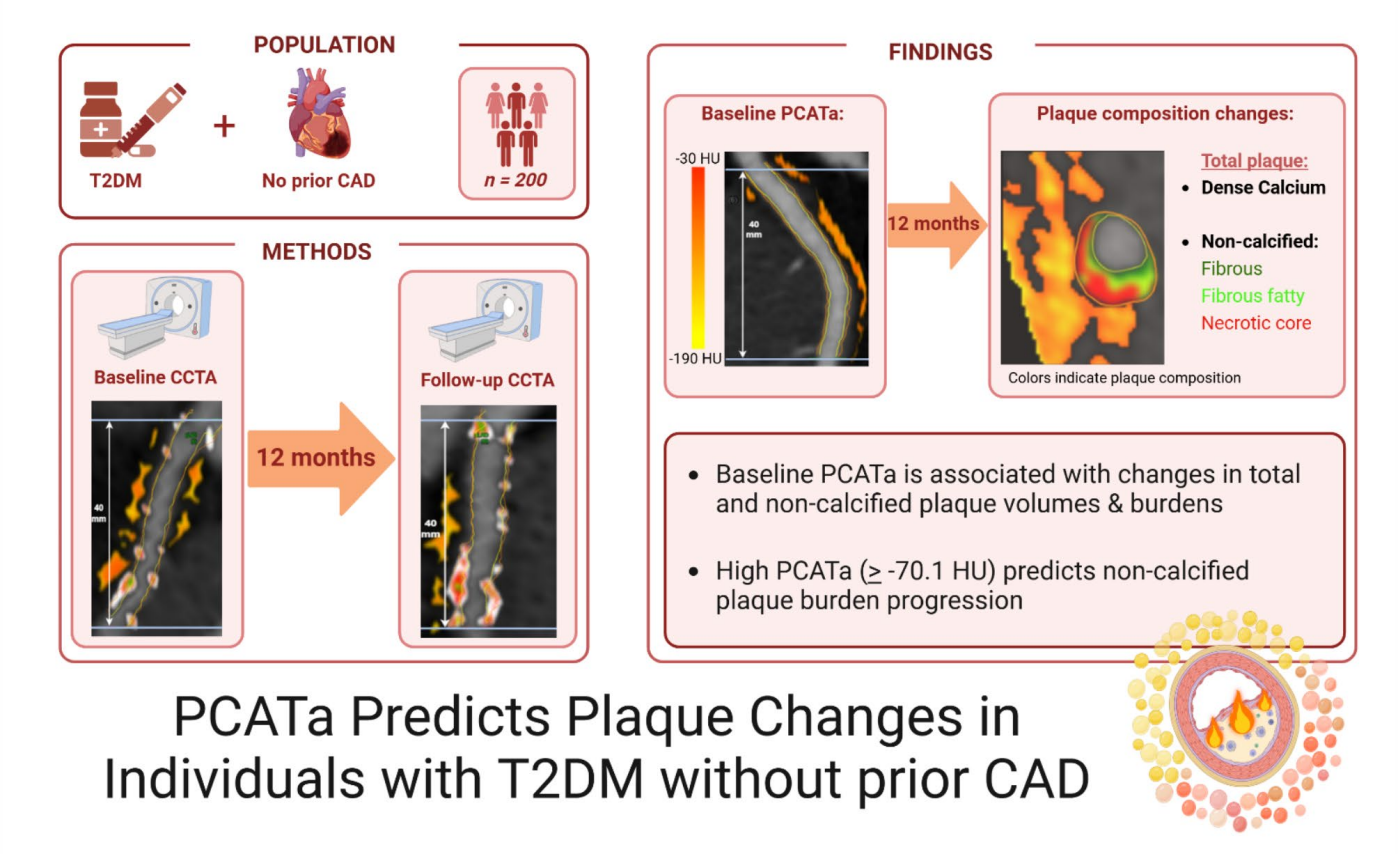

**Research insights:**

What is currently known about this topic?

1. Type 2 diabetes mellitus (T2DM) and coronary artery disease (CAD) share inflammatory mechanisms.
2. Individuals with T2DM face a two- to four-fold increased risk of CAD compared with those without T2DM.
3. Pericoronary adipose tissue attenuation (PCATa) is a novel marker of coronary inflammation.

What is the key research question?

- Can baseline PCATa predict compositional plaque changes over 12 months in T2DM without known CAD?

What is new?

1. Baseline PCATa relates to higher total and non-calcified plaque (NCP) volumes after adjustment.
2. Baseline PCATa associates with increased total- and NCP burden after multivariable adjustment.
3. High baseline PCATa (> -70.1 HU) independently predicts NCP burden progression.

How might this study influence clinical practice?

- PCATa may be a marker for subclinical atherosclerosis progression.

**Supplementary Information:**

The online version contains supplementary material available at 10.1186/s12933-025-02694-9.

## Background

Type 2 diabetes mellitus (T2DM) and coronary artery disease (CAD) are major global health burdens that significantly contribute to mortality and economic costs [1, 2]. The risk of developing CAD is two to four times higher in individuals with T2DM compared with those without diabetes [2, 3], likely driven by shared inflammatory pathways [4]. Individuals with T2DM often exhibit chronic systemic low-grade inflammation, endothelial dysfunction, and metabolic disturbances, which contribute to the accelerated development of CAD [5]. Early identification and management of CAD in individuals with T2DM are critical to reduce this burden.

Pericoronary adipose tissue attenuation (PCATa) can be measured using coronary computed tomography angiography (CCTA) and has recently been identified as a promising non-invasive marker of coronary inflammation [6]. PCATa reflects the local inflammatory processes in the adipose tissue surrounding the coronary arteries, and it has been associated with CAD presence [6], high-risk plaque features [7], non-calcified plaque (NCP) progression [8], and cardiovascular mortality [9]. PCATa has been studied in relation to T2DM, showing elevated levels in patients with T2DM compared to those without [10]. PCATa was also found to be significantly higher in patients with T2DM who experienced cardiovascular events than in those without events [11]. However, the predictive value of PCATa for plaque changes and progression over time in high-risk individuals, particularly those with T2DM, remains uncertain.

The aim of this study was to investigate whether participant-level baseline PCATa could predict changes in compositional plaque volumes and burden over 12 months in individuals with T2DM without symptoms or known CAD.

##  Methods

###  Study design

This was a single-center, prospective longitudinal study including participants with T2DM, who had no symptoms or known CAD. Participants completed a baseline visit at the Cardiovascular Research Unit at Odense University Hospital, Svendborg, between March 2016 and September 2017, followed by a 12-month follow-up visit.

This study was a post-hoc analysis of data collected within the CARPE-DIEM trial [12]. We conducted the study in compliance with the principles of the Declaration of Helsinki and received approval from the Regional Committees on Health Research Ethics for Southern Denmark (ID S-20150029) and the Danish Data Protection Agency (ID 2008-58-0035). The current post-hoc analysis was registered at ClicalTrials.gov (protocol ID: NCT06644651).

### Study population

We recruited participants with T2DM from the Endocrinology Outpatient Clinic and the Retina Photography Clinic at Odense University Hospital, Svendborg. All participants underwent CCTA for research purposes only, with no clinical indication for the examination.

#### Criteria for participation

**Fig. 1:**
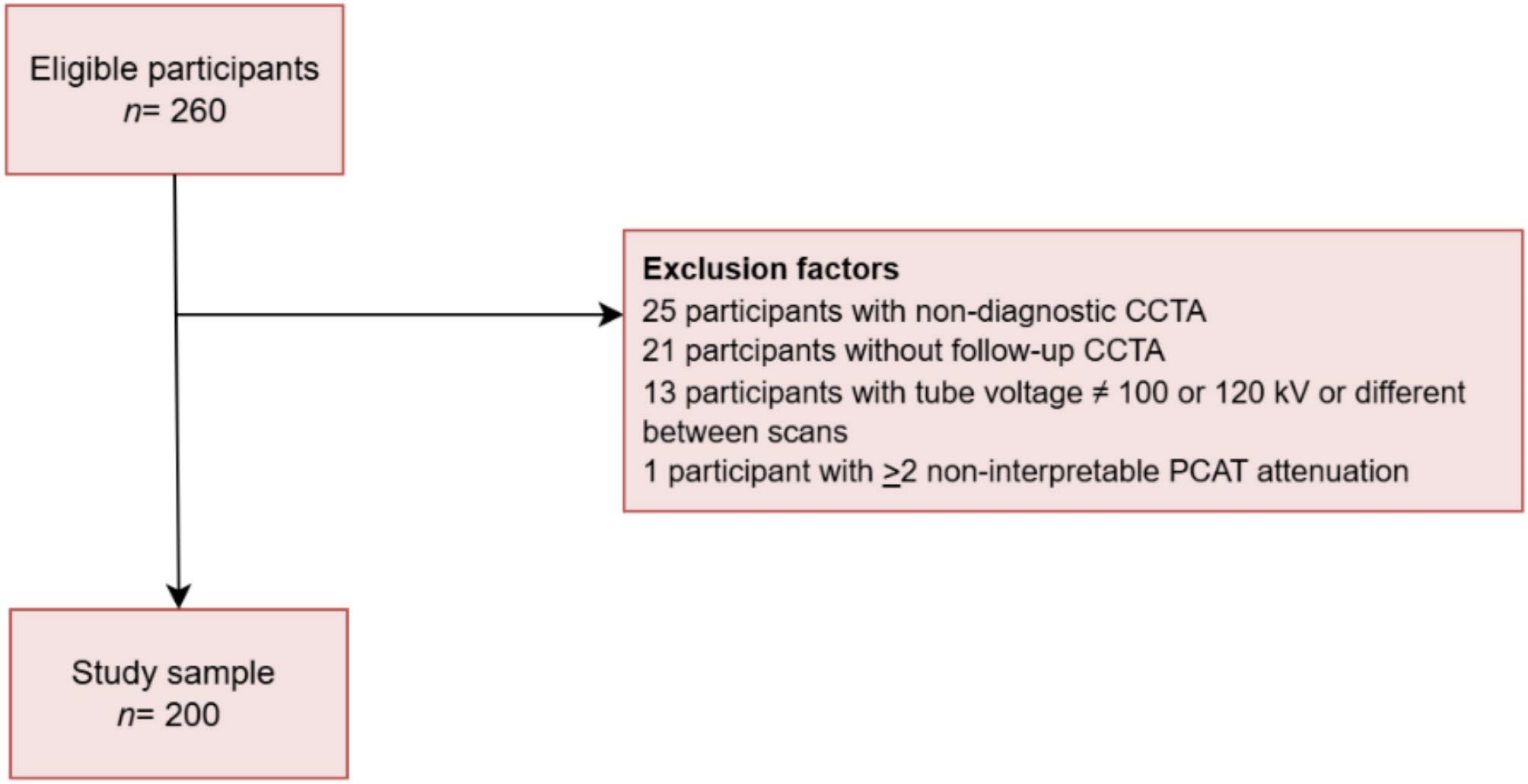
CONSORT diagram. This flowchart illustrates the study sample selection, including their eligibility based on exclusion criteria related to scan analysis. *n* = numbers; CCTA = Coronary computed tomography angiography; kV = Kilo Volt; PCAT = Pericoronary adipose tissue

A total of 260 participants underwent CCTA (Fig. 1). Inclusion criteria were:

 [1] Age *≥* 18 [2], ability to provide informed consent, and [3] a confirmed diagnosis of T2DM.

Exclusion criteria were: [1] A prior CAD diagnosis [2], symptoms of CAD, including angina or radiating chest pain [3] documented heart failure [4], reduced kidney function with an estimated glomerular filtration rate (eGFR) < 45 ml/min, or [5] a known allergy to contrast agents.

During CCTA assessments, 60 scans were excluded for the following reasons: [6] Non-diagnostic CCTA, which included severe motion artefacts, inadequate image quality, coronary abnormalities, non-contrast scans, analysis limited to only one vessel [7], no follow-up CCTA [8], more than two non-interpretable PCATa measurements, or [9] tube voltages that either were outside the range of 100–120 kilo Volt (kV) or did not match between the baseline and follow-up CCTA scans.

#### Rationale for exclusion

Tube voltage is considered to affect the measurements of both PCATa [13] and plaque composition [14]. Demographic data for the excluded participants are presented in supplementary Table 1.

###  Baseline assessments

At the baseline visit, we recorded a detailed health status for all participants [12]. Written and oral consent were obtained before the examination. We used a questionnaire to assess cardiovascular risk factors, including age, smoking status, medical history, and medication use. Tobacco exposure was evaluated in terms of pack years (one pack year = 20 cigarettes per day for one year). We measured height, weight, and waist circumference at the narrowest point in the abdomen and hip circumference at the widest part of the buttocks. The waist-hip-ratio was calculated by dividing the waist circumference by the hip circumference. Blood pressure was measured in a seated position after 10 min rest. Participants underwent annual screening for retinopathy and neuropathy as part of their routine clinical controls. Retinopathy was graded from zero to four, with a grade of one or higher indicating the presence of retinopathy. Peripheral neuropathy was classified based on reduced or absent vibratory sensation. 

 Blood samples were analyzed for glycated hemoglobinA1c (HbA1c), total cholesterol, high-density lipoprotein (HDL), low-density lipoprotein (LDL), triglycerides, creatinine, eGFR, and C-reactive protein (CRP). We collected spot urine to assess for albuminuria and to estimate the urine albumin to creatinine ratio (UACR). We classified participants with an UACR value above 30 mg/g as having nephropathy.

We defined diabetic complications as the presence of either nephropathy, retinopathy and/or neuropathy, specifying the total number of complications.

### CCTA acquisition

All participants underwent CCTA at the baseline visit and after 12-month follow-up using the same CCTA 256-slice scanner (GE Healthcare, Revolution, Milwaukee, WI, USA). Participants were prepared with a 7.5 mg tablet of Ivabradine the evening before the CCTA and again on the morning of the procedure. To dilate the coronary arteries, all participants received one dose of sublingual nitroglycerin spray (0.4 mg/dose) 2–5 min before the CCTA. If the heart rate exceed 65 bpm, 5 mg intravenous beta-blocker, up to a maximum of 30 mg, was administered. Images were acquired by electrocardiogram-gated prospective acquisition at 75% of the R-R interval, with an additional padding of 45 milliseconds for reconstruction. For participants with high heart rates, an additional phase was acquired at 40% phase of the R-R interval, and a repeated scan was acquired if the heart rhythm was irregular. First, an unenhanced scan was performed to estimate coronary calcium score, utilizing the scoring system described by Agatson et al. [15]. Following this, a contrast-enhanced coronary artery scan was performed to evaluate plaque presence and characteristics. A total of sixty milliliters (mL) of iodine contrast (Visipapque 320 mg/mL) was injected at 5 mL/second, with scan timing synchronized to maximal attenuation in the ascending aorta. Tube voltage was adjusted based on body size, ranging from 80 to 140 kV, while the tube current varied between 150 and 700 mA. The slice thickness and reconstruction interval were both 0.625 mm. Adaptive statistical iterative reconstruction (ASiR) at 40% was applied to enhance image quality. All available phases were reconstructed, and the highest quality images were selected for analysis. The median radiation dose per scan was 2.01 millisievert.

### Image analysis

We analyzed CCTA images using the validated software program QAngioCT Research Edition version 3.2.0.13 (Medis Medical Imaging, Leiden, NL) [16]. Experienced observers (TRA and LJH) conducted the analysis blinded to patient characteristics. Repeatability was high for total plaque volume, with intra-observer (*r* = 0.98) and inter-observer (*r* = 0.91) correlations, as reported in [12], which also presents repeatability for additional plaque components. The software automatically generated centerlines for each coronary vessel, and we manually adjusted the longitudinal and cross-sectional lumen and vessel contours when necessary. Automated segmentation was performed by the software and manually corrected as needed following the 16-segment coronary artery tree model [17]. All segments underwent visual examination to detect the presence of plaques, defined as structures *≥* 1mm^3^ within or adjacent to the lumen and visible in *≥* two planes. The plaque component was measured on a per-participant level. The plaque volumes were determined by automated calculation of the total plaque volume (mm^3^) based on fixed Hounsfield unit (HU) thresholds. The total plaque volume was further sub classified into compositional volumes using predefined HU cut-off values [18]: (1) NCP (-30 to 350 HU) and (2) Calcified plaque (CP) (> 351 HU). The plaque burden were quantified as the normalized atheroma volume to adjust for differences in vessel length and was defined as the vessel volume minus the lumen volume divided by the segment length (unit: mm^3^/mm) [19]. The total, NCP, and CP volumes were normalized using this formula.

###  PCATa measurement

**Fig. 2:**
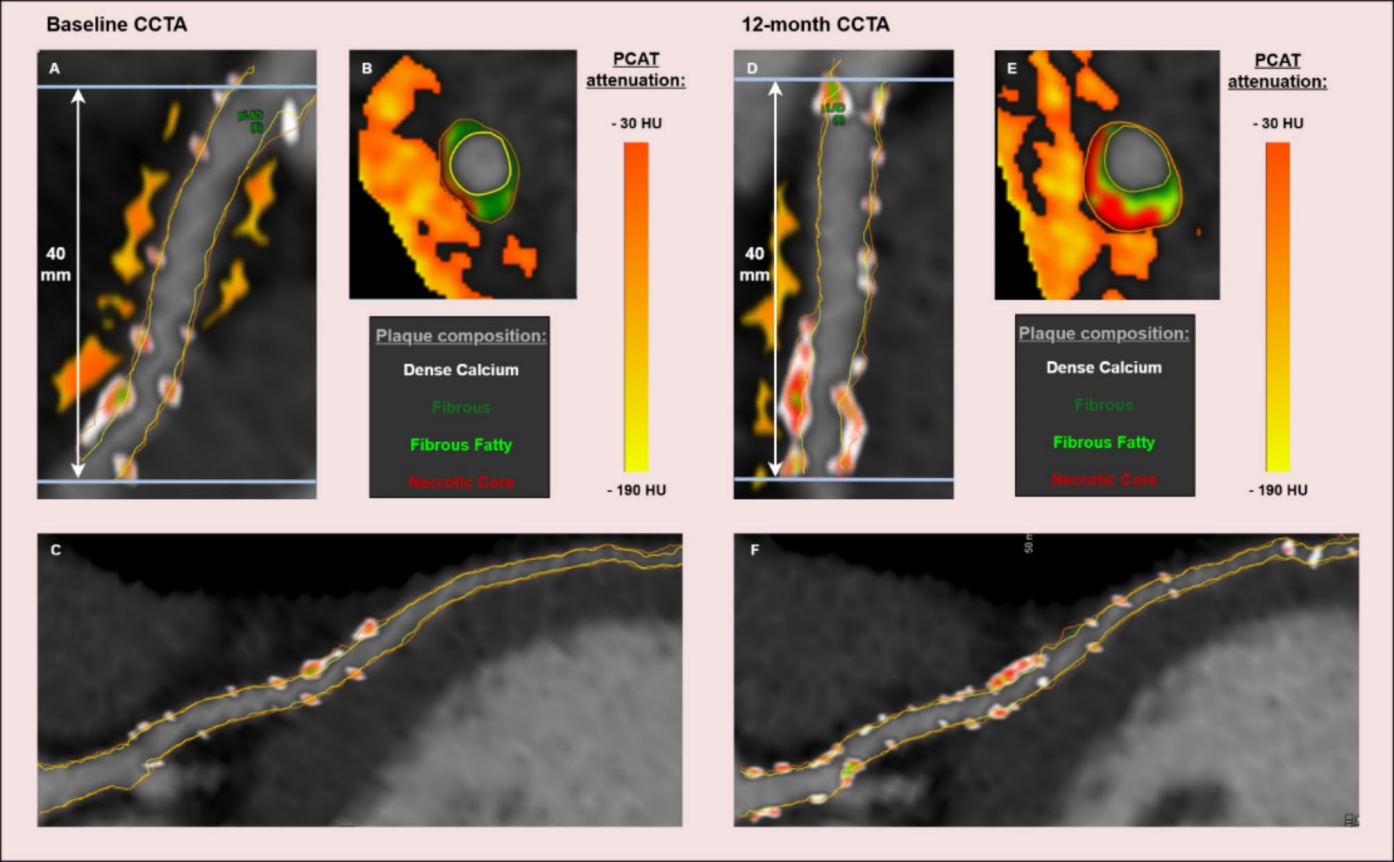
Case example of a 51-year-old male with a history of hypertension, hyperlipidemia, and type 2 diabetes for 11 years. The participant-level PCATa was − 68.0 HU at baseline and − 68.4 HU at 12 months. Progression in non-calcified plaque volume was quantified as + 194 mm³, corresponding to an increase of + 0.9 mm³/mm in non-calcified plaque burden.

PCATa was measured with the QAngioCT Research Edition version 3.2.0.13 (Medis Medical Imaging, Leiden, NL) software. Experienced observers (KSO and IIAB) measured PCATa around all three major coronary arteries, the RCA (Right Coronary Artery), LAD (Left Anterior Descending), CX (Circumflex). PCATa refers to the adipose tissue located within a radial distance from the outer vessel wall equal to the diameter of the coronary vessel, with an attenuation range of -190 to − 30 HU (Fig. 2) [6, 20]. We measured PCATa around the proximal 40 mm segments of the LAD and CX, starting after branching from the left main artery, while RCA measurements were done from the 10th -50th mm from the ostium to minimize interference from the aortic wall. PCATa values from the three vessels were averaged to derive the participant-level measurement [21]. The participant-level PCATa demonstrated strong correlations across all vessels (Fig. S1). To account for differences in attenuation between scans done at different tube voltages, scans at 100 kV were adjusted using a validated conversion factor of 1.11485, ensuring comparability with scans at 120 kV [6].

Quantified plaque composition and PCATa are shown for baseline (A-C) and follow-up (D-F). Plaque composition is represented using color codes corresponding to specific tissue types based on HU values: white = dense calcium, dark green = fibrous, light green = fibrous fatty, and red = necrotic core (A-F). PCATa is visualized using an adipose tissue HU color scale depicted in the accompanying bars (A-B, D-E). (A) Curved multiplanar view of the 40-mm proximal segment of the LAD, showing quantification of PCATa and plaque composition at baseline. (B) Cross-sectional view of plaque composition and PCATa at baseline. (C) Curved multiplanar view of the entire LAD at baseline. (D) Curved multiplanar view of the 40-mm proximal segment at follow-up. (E) Cross-sectional view of plaque composition and PCATa at follow-up. (F) Curved multiplanar view of the entire LAD at follow-up.

### Statistics

We performed statistical analysis using Stata 18.0 software (StataCorp, 2023, College Station). Baseline data were summarized using descriptive statistics: counts (*n*) and percentages *(%)*, means with standard deviations (± SD), or medians with interquartile ranges (IQR) based on data distribution. We tested normality using the Shapiro-Wilk test and visually inspected with quantile-quantile plots and histograms. The Wilcoxon rank test or paired t-test were used to compare baseline PCATa, plaque volumes, and burden with 12-month data, depending on data distribution. Multivariable linear regression analyses explored associations between baseline PCATa and changes in plaque volumes and burden. Log-transformation was applied where necessary to improve normality. Baseline characteristics with p-values < 0.05 in univariable analysis were considered for the multivariable model. The Wald Test assessed the significance of variables, retaining those with p-values < 0.05, while clinically relevant confounders were included regardless of univariable p-values. The final regression model included sex, age, CRP and triglycerides at baseline, and baseline use of statins and liraglutide. We categorized PCATa as low or high basedon a cut-off of -70.1 HU, as defined in the CRISP-CT study [9]. An unpaired t-test was conducted to compare compositional changes in plaque burden between the low and high PCATa groups, and the results were visualized with boxplots. A multivariable logistic regression analysis evaluated the associations of risk factors, including high PCATa, in predicting NCP burden progression, which was treated as a binary variable (NCP progression: yes/no). Statistical significance was defined as *p* < 0.05, and 95% confidence intervals were reported.

## Results

### Clinical characteristics

The study included 200 participants with T2DM who had neither symptoms nor known CAD (Table 1). Participants had a mean age of 61 years (± 9.4) and 72% were male. The mean body mass index (BMI) was 30.5 kg/m² (± 4.5), with a waist-hip-ratio of 1.0 (± 0.09). Medication use reflected the comorbidities of the participants, with 69% taking antihypertensive medications, 75% on statins, and 83% on metformin. Other diabetes treatments included liraglutide (26%), dipeptidyl peptidase-4 inhibitors (15%), sulfonylurea (18%), sodium-glucose transport inhibitor (SGLT2i) (9%), and insulin (39%). Participants had a mean diabetes duration of 10 years (± 6), with 28% having nephropathy, 24% retinopathy, and 28% neuropathy. Nearly half had no diabetic complications, while 33% had one, and 18% had two or more. Biochemically, HbA1c averaged 60 ± 14 mmol/mol, LDL was 2.0 ± 0.8 mmol/L, the median UACR was 12 (6–37) mg/g, and the kidney function was preserved with an eGFR of 90 (89–90) mL/min/1.73 m². The median coronary calcium score was 74 (1-423), and the mean baseline PCATa − 74.9 ± 6 HU. The distribution of baseline PCATa is illustrated in Fig. S2.

**Table 1:** Participant characteristics

Demographic data	*n* = 200
Age, years	61 ± 9.4
Sex, male	153 (72)
BMI, kg/m^2^	30.5 ± 4.5
Waist-hip-ratio	1.0 ± 0.09
Systolic BP, mmHg	141 ± 16
Diastolic BP, mmHg	86 ± 10
Never smoker	78 (39)
Former smoker	75 (38)
Active smoker	47 (24)
Pack years	9 (0–30)
Medications	
Antihypertensive medication	137 (69)
Statins	150 (75)
Metformin	165 (83)
Liraglutide	51 (26)
SGLT2i	18 (9)
Insulin	77 (39)
DPP4i	30 (15)
Sulfonylurea	36 (18)
Diabetes-related	
Diabetes duration, year	10 ± 6
Neuropathy (35 missing)	47 (28)
Retinopathy (7 missing)	47 (24)
Nephropathy	56 (28)
Number of DM complications	
*0*	98 (49)
*1*	66 (33)
*≥* *2*	35 (18)
Biochemistry	
HbA1c, mmol/mol	60 ± 14
Total cholesterol, mmol/L	4.1 ± 1.0
HDL, mmol/L	1.1 (1-1.4)
LDL, mmol/L	2.0 ± 0.8
Triglycerides, mmol/L	1.8 (1.3–2.5)
eGFR, mL/min/1.73m^2^	90 (89–90)
UACR, mg/g	12 (6–37)
CRP, mg/L	1.5 (0.6–3.5)
CCTA	
CCS	74 (1-423)
PCATa, HU	-74.9 ± 6

Values are mean ± standard deviation (SD), median + interquartile range (IQR), or counts (*n*) + proportions (*%*). BMI = body mass index; BP = blood pressure; SGLT2i = sodium-glucose cotransporter-2 inhibitor; DPP4i = dipeptidyl peptidase-4 inhibitor, DM = diabetes mellitus; HbA1c = glycated hemoglobin A1C; HDL = high-density lipoprotein; LDL = low-density lipoprotein; eGFR = estimated glomerular filtration rate; UACR = urinary albumin-to-creatinine ratio; CRP = C-reactive protein, CCTA = Coronary computed tomography angiography, CCS = Coronary calcium score, PCATa = Pericoronary adipose tissue attenuation.

### CCTA changes over 12 months

**Fig. 3:**
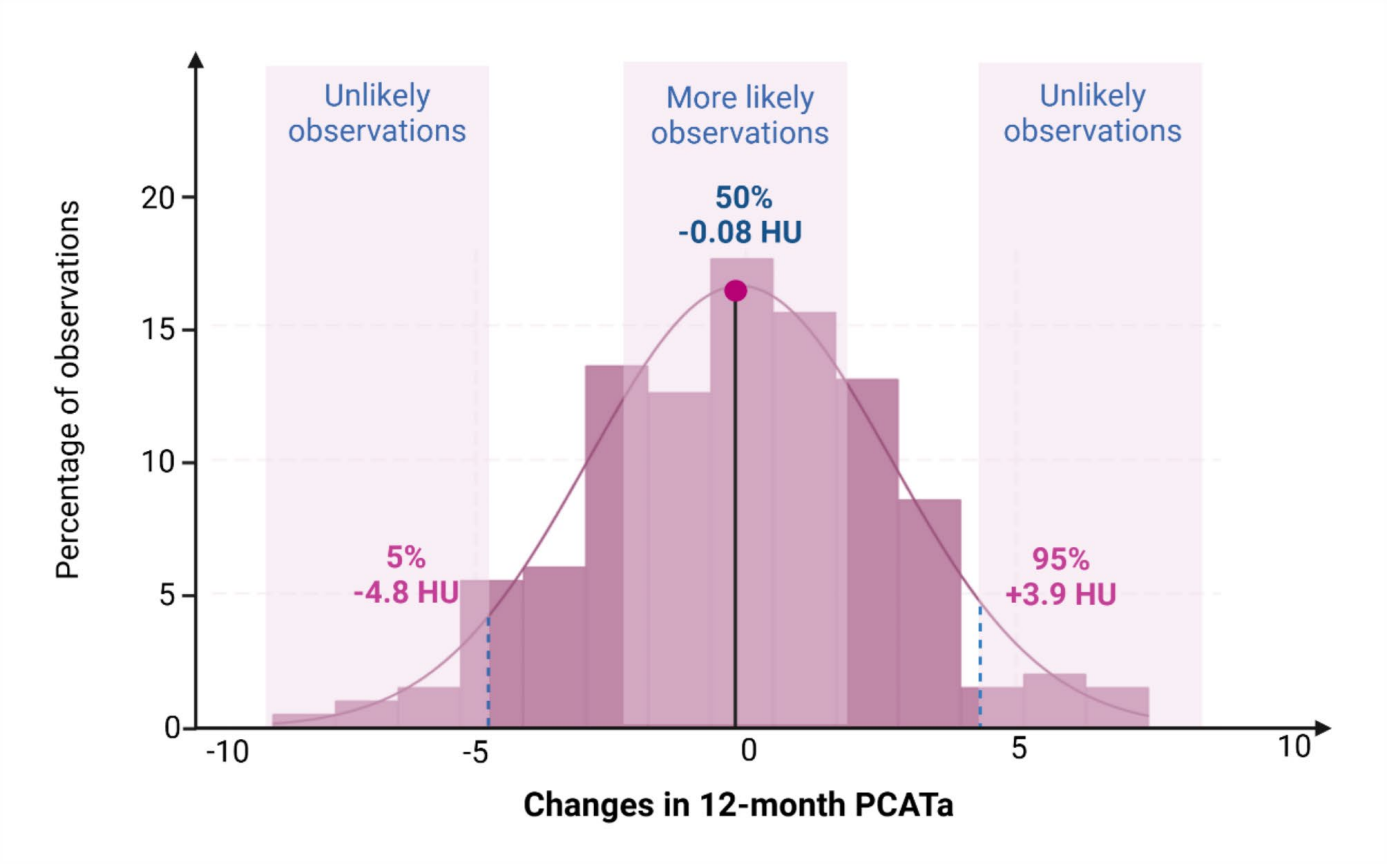
Histogram showing changes in PCATa over 12 months

Table 2 presents the CCTA findings at baseline and 12-month follow-up, displaying changes observed over the study period. CCTA findings showed an increase in total plaque volume (Δ + 39 mm³, *p* = 0.03), driven by a significant rise in calcified plaque (Δ + 19 mm³, *p* < 0.0001), while NCP changes were non-significant (Δ + 17 mm³, *p* = 0.1). Total plaque burden increased (Δ + 0.2 mm³/mm, *p* = 0.001) with significant changes in CP burden (Δ + 0.08 mm³/mm, *p* < 0.0001), and changes in NCP burden of similar magnitude (Δ + 0.09 mm³/mm, *p* = 0.05) but borderline statistical significant. PCATa remained stable (*p* = 0.6). Figure 3 illustrates the distribution of changes in PCATa over 12 months, showing a median change of -0.08 HU, with the 5th percentile of - 4.8 HU and the 95th percentile of + 3.9 HU.

**Table 2:** Changes in CCTA findings over 12 months

	Baseline	Follow-up	Positive changes (*n*)	Negative changes (*n*)	*p*-value BL vs. FU	Δ Mean change
PCATa (HU)	− 74.9 (± 6.0)	-75.0 (± 6.1)	98	102	0.6	-0.1
Plaque volumes, mm^3^						
Total	972 (741–1341)	985 (767–1392)	109	91	0.008	+ 39
Calcified	76 (21–208)	84 (29–242)	135	65	< 0.0001	+ 19
Non-calcified	845 (696–1070)	851 (700–1059)	105	95	0.1	+ 17
Plaque burden, mm^3^/mm						
Total	3.03 (2.35–4.01)	3.08 (2.35–4.36)	112	88	0.001	+ 0.2
Calcified	0.23 (0.07–0.64)	0.26 (0.09–0.75)	137	63	< 0.0001	+ 0.08
Non-calcified	2.64 (2.13–3.11)	2.61 (2.15–3.20)	106	94	0.05	+ 0.09

Means ± SD or medians (IQR) are reported based on data distribution to illustrate changes. Statistical tests (paired t-test or Wilcoxon rank test) were applied accordingly. PCATa = Pericoronary adipose tissue attenuation. CCTA = Coronary computed tomography angiography, HU = Hounsfield Unit, n = numbers, BL = Baseline, FU = follow-up.

The graph illustrates the distribution of changes in PCATa, with key percentiles marked: 5%, 50%, and 95%. HU = Hounsfield Unit, PCATa = Pericoronary adipose tissue attenuation. Created in BioRender. Lambrechtsen, J. (2025). https://BioRender.com/w04x482.

### Association between baseline PCATa and changes in plaque volumes and burden

In the univariable analysis (Table 3), baseline PCATa was significantly associated with changes in total plaque volume (β = 0.004, *p* = 0.02), NCP volume (β = 0.005, *p* = 0.03), total plaque burden (β = 1.5, *p* = 0.02), and NCP burden (β = 1.8, *p* = 0.02). In the multivariable analysis, adjusted for sex, age, CRP and triglycerides-levels at baseline, and baseline use of: statins and liraglutide, these associations remained significant. Baseline PCATa was associated with increases in total plaque volume (β = 0.005, *p* = 0.005), NCP volume (β = 0.006, *p* = 0.007), total plaque burden (β = 1.7, *p* = 0.007), and NCP burden (β = 2.0, *p* = 0.006). No significant associations were observed for CP volume or burden in either model.

**Table 3:** Uni- and multivariable regression analysis of changes in plaque compositions by baseline PCATa

	Univariable		Multivariable	
	β	p-value	β	p-value
Δ Plaque volumes (mm^3^)				
Δ Total	0.004	0.02	0.005	0.005
Δ Calcified_*_	0.04	0.3	0.04	0.3
Δ Non-calcified	0.005	0.03	0.006	0.007
Δ Plaque burden (mm^3^/mm)				
Δ Total	1.5	0.02	1.7	0.005
Δ Calcified_*_	0.002	0.4	0.002	0.4
Δ Non-calcified	1.8	0.02	2.0	0.005

The multivariable analysis was adjusted for sex, age, and the following variables at the baseline visit: use of statins and liraglutide, CRP, triglycerides. Δ = changes over 12-months. _*_ Log-transformed

###  Association of high PCATa with changes in plaque burden

**Fig. 4:**
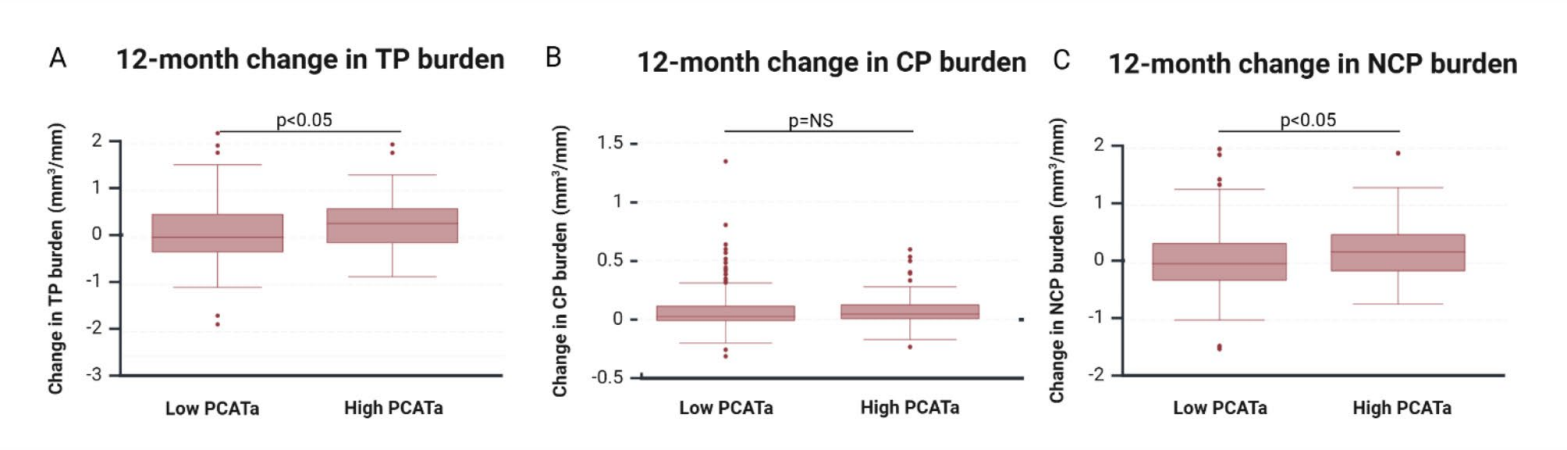
Boxplots for compositional changes in plaque burden by low versus high PCATa

Participants were categorized into low and high groups based on baseline PCATa levels, using the– 70.1 HU cut-off from the CRISP-CT study [9]. High PCATa was observed in 44 (22%) participants, with demographic data for the low vs. high PCATa groups provided in supplementary Table 2. High baseline PCATa was significantly associated with changes in total plaque burden (0.35 ± 0.6 mm^3^/mm, *p* = 0.04) (Fig. 4A) and NCP burden (0.25 ± 0.5 mm^3^/mm, *p* = 0.03) (Fig. 4C), but no significant difference was found for changes in CP burden (0.09 ± 0.2 mm^3^/mm, *p* = 0.6) (Fig. 4B) (supplementary Table 3).

Low PCATa < -70.1 HU. High PCATa *≥* -70.1 HU. PCATa = Pericoronary adipose tissue attenuation. TP = Total plaque, HU = Hounsfield unit, NCP = Non-calcified plaque, CP = Calcified plaque. Created in BioRender. Lambrechtsen, J. (2025) https://BioRender.com/p26m229.

###  Prediction of NCP burden progression by high baseline PCATa

A multivariable logistic regression analysis evaluated the associations between various cardiovascular risk factors and NCP burden progression. The model included high baseline PCATa (*≥* -70.1 HU), sex, age, waist-hip-ratio, baseline use of statins and liraglutide, CRP- and triglyceride levels at baseline, and baseline NCP burden. High baseline PCATa was found to be independently associated with an increase in NCP burden (odds ratio (OR) 3.5, 95% CI 1.6–7.7; *p* = 0.002) (Table 4). Notably, high PCATa was the only covariate significantly associated with NCP burden progression.

**Table 4:** Multivariable logistic regression analysis of risk factors associated with progression of NCP burden

	Odds ratio	95% CI	*P*-value
PCATa *≥* -70.1 HU	3.5	1.6–7.7	0.002*
Sex	0.7	0.3–1.7	0.5
Baseline NCP burden	0.9	0.6–1.3	0.5
Age	1.0	0.9-1.0	0.7
Waist-hip-ratio	28	0.1–2050	0.1
Statin use	1.0	0.5–2.1	0.9
Liraglutide	0.7	0.3–1.4	0.3
CRP	1.1	1.0-1.2	0.2
Triglycerides	1.0	0.8–1.3	0.9

NCP burden progression was treated as a binary outcome (Yes/No) in the logistic regression analysis.

NCP = Non-calcified plaque, PCATa = Pericoronary adipose tissue attenuation, HU = Hounsfield unit, CRP = C-reactive protein.

## Discussion

In this study, we investigated whether participant-level baseline PCATa could predict changes in compositional plaque volumes and burden over 12 months in individuals with T2DM without symptoms or known CAD. Our key findings were as follows: (1) Plaque compositional volumes and burden increased over 12 months, while PCATa remained stable. (2) Baseline PCATa was significantly associated with total and non-calcified plaque volumes and burden after adjustment, but not with calcified plaque volume or burden. (3) High baseline PCATa (*≥* -70.1 HU) was an independent predictor of non-calcified plaque burden progression.

### Inflammatory mechanisms in T2DM and CAD

Type 2 diabetes is characterized by insulin resistance and systemic low-grade inflammation, probably driven by dysfunction of adipose tissue. This dysfunction leads to an excessive production of pro-inflammatory cytokines, such as interleukin-6 (IL-6) and tumor necrosis factor-alpha (TNFα), while simultaneously reducing anti-inflammatory adipokines (i.e. adiponectin) [5]. In response to elevated TNFα levels, the liver produces high-sensitivity CRP (hs-CRP), which is a widely used marker of systemic low-grade inflammation. A prospective cohort study of 1,679 patients with T2DM used hs-CRP as a marker of systemic low-grade inflammation. They found that hs-CRP was independently associated with increased vascular- and all-cause mortality but not with myocardial infarction [22]. These findings highlight the role of systemic low-grade inflammation in T2DM-related complications but suggest that circulating hs-CRP may not fully capture local vascular inflammation within the coronary arteries. This supports the notion that vascular inflammation itself, rather than systemic markers, is the primary driver of coronary atherosclerosis through paracrine signaling [6]. Inflammatory cytokines released from the inflamed coronary arteries into the surrounding PCAT induce local lipolysis and inhibit adipogenesis, resulting in pericoronary edema and reduced lipid accumulation and adipocyte volume [6, 23]. These compositional changes in PCAT can be detected on CCTA as increased attenuation [6]. Interestingly, neither Dai et al. [24] nor Goeller et al. [25] could identify an association between PCATa and circulating hs-CRP. However, Goeller et al. found that RCA-PCATa was an independent predictor of major adverse cardiovascular events (MACE) (HR 2.01, *p* = 0.04). These findings suggest that PCATa reflects local coronary inflammation distinct from systemic inflammation. In our study, baseline CRP was not a significant predictor of NCP progression after 12 months (OR 1.1, *p* = 0.2) whereas high PCATa were independently associated with NCP burden progression (OR 3.5, *p* = 0.002).

### PCATa in T2DM

PCATa has been found to be significantly higher in individuals with T2DM compared with those without, with one study reporting a mean increase of five HU in the RCA, irrespective of high-risk plaque features or stenosis degree [10]. Another study integrated PCATa into a comprehensive risk factor model to improve the diagnostic efficacy of detecting CAD in patients with T2DM [26]. This model combined clinical risk factors with CT-based parameters, and the addition of PCATa significantly improved its ability to identify CAD. RCA-PCATa was found to be an independent indicator of CAD [26]. Furthermore, PCATa has demonstrated prognostic value in identifying high-risk patients with CAD. Ichikawa et al. [11] studied more than 300 patients with T2DM who underwent clinically indicated CCTA and were followed for 4 years. Their findings revealed that LAD-PCATa was significantly higher in patients who experienced cardiovascular events compared with those who did not (-68.5 ± 6.5 HU vs. -70.8 ± 6.1 HU, *p* = 0.045). Similarly, elevated lesion-specific PCATa independently predicted MACE in patients with T2DM followed over a 4-year period [27]. Consistent with these findings, our study showed that baseline PCATa was significantly associated with changes in total and NCP volumes and burden over 12 months, even after adjusting for relevant confounders. Our study stands out by calculating PCATa across all three major coronary arteries as a participant-level measure. To our knowledge, this is the first study to investigate participant-level PCATa in a population with T2DM. Previously, we discussed the variability in measurement methods and approaches for assessing PCATa [28]. Ma et al. further elaborate on the different PCATa methodologies in their review [21].

### Targeting inflammation indicated by PCATa in T2DM

PCATa shows promise in monitoring treatment effects in patients with T2DM. Recent studies highlight the impact of different treatment strategies on reducing coronary inflammation in this high-risk population. A prospective study demonstrated that 48 weeks of evolocumab (a PCSK9 inhibitor) treatment reduced NCP volume, necrotic plaque volume, and high-risk plaque features. It also decreased PCATa, partly mediated through reductions in lipoprotein(a) [29]. Conversely, poor glycemic control and inadequate diabetes management have been associated with elevated PCAT attenuation, whereas metformin and acarbose appear to reduce PCATa, indicating their potential to modulate coronary inflammation [30]. Similarly, the glucagon-like peptide-1 receptor agonist (GLP1-RA), semaglutide, has been associated with reduced PCATa in patients with T2DM, suggesting that semaglutide possesses anti-inflammatory properties that may modulate coronary inflammation [31]. In our study, the use of liraglutide, a GLP1 RA, and statins at baseline were included as clinically relevant treatments, with the Wald test showing that both significantly decreased PCATa.

### PCATa as a predictor of plaque vulnerability

Previous studies suggest that PCATa can predict progression of plaque vulnerability. A PARADIGM sub-study from Lee et al. [32] found that increase in PCAT density, measured at the lesion-level, was associated with total plaque volume progression, primarily driven by fibrous plaque, a component of NCP. Similarly, Goeller et al. [8] reported that NCP burden changes were associated with PCATa changes. Furthermore, they found that baseline PCATa independently predicted NCP burden progression. These results aligns with our findings that baseline PCATa could predict changes in total and NCP volumes and burden after 12 months. We also found that a high baseline PCATa (*≥* -70.1 HU) independently predicted NCP burden progression similar to Goeller et al. Notably, we did not find a relationship between changes in PCATa and compositional plaque volumes, our findings suggested that ΔPCATa was stable over 12 months (median change: -0.08 HU) despite significant increases in plaque volumes and burden. This discrepancy may relate to our shorter follow-up, as we only followed participants for 12 months, compared to 3.4-year follow-up by Goeller et al. [8] and more than 2-years scan intervals in Lee et al. [32]. Additionally, both of their study populations consisted of patients with stable CAD, while our population included individuals with T2DM and no symptoms or history of CAD, this might explain the stability of PCATa observed in our T2DM population. Another possible explanation is the effect of treatment, as the majority of our population was well-medicated for diabetes and their comorbidities, 69% were on antihypertensive medications, 75% on statins, and 83% on metformin, which may have helped maintain a steady state of vascular inflammation.

Our study found no associations between baseline PCATa and changes in CP burden, consistent with Goeller et al. [8] and Lee et al. [32]. In a prior cross-sectional study, we even observed lower PCATa with greater CP burden [28]. This supports the hypothesis that calcification may not drive plaque inflammation, though its exact role remains uncertain.

###  Methodological implications

This study has several strengths, including its prospective longitudinal design with a 12-month follow-up, which allows for the assessment of temporal changes. However, as a single-center study, the findings may not be fully generalizable to broader populations. The relatively small sample size of 200 participants may not fully capture the heterogeneity of broader populations, and the limited number of individuals in the high PCATa group should be considered when interpreting the results. Additionally, our cohort consisted of participants with T2DM and without symptoms or known CAD, who were relatively well-medicated for their diabetes and comorbidities. This may limit the generalizability of our findings to populations with established CAD, who may exhibit different plaque characteristics and disease progression. Expanding future research through multicenter collaborations with larger cohorts could improve the generalizability of the results.

As shown in Supplementary Table 1, the higher BMI among excluded participants is likely due to tube voltage exclusions, which are body size-dependent. However, their lower statin use may introduce selection bias, which should be considered in the interpretation of the results.

A key strength of this study is the detailed collection of participant characteristics. Furthermore, we utilized a validated software program and experienced observers for the assessment of plaque and PCATa, ensuring robust and accurate data interpretation. However, manual adjustments during image analysis may introduce some variability. Additionally, technical factors, such as imaging protocols and individual participant differences, may influence the quantification of PCATa. Addressing technical considerations in future studies could further refine and standardize PCATa measurements.

## Conclusion

This study demonstrates the potential of PCATa as a participant-level biomarker for assessing plaque changes in individuals with T2DM without symptoms or known CAD. Baseline PCATa was significantly associated with increases in total and non-calcified plaque volumes and burden over 12 months but was not associated with calcified plaque changes. High PCATa levels (*≥* -70.1 HU) independently predicted non-calcified plaque burden progression supporting its potential role as a marker of subclinical atherosclerosis progression. Future studies should validate the clinical utility of PCATa in high-risk populations, assess its prognostic value for long-term cardiovascular outcomes, and explore its role in guiding therapeutic interventions.

## Electronic supplementary material

Below is the link to the electronic supplementary material.

Supplementary Material 1

## Data Availability

No datasets were generated or analysed during the current study.

## Abbreviations

PCATa: Pericoronary adipose tissue attenuation
T2DM: Type 2 diabetes mellitus
CAD: Coronary artery disease
NCP: Non-calcified plaque
CCTA: Coronary computed tomography angiography
CAD: Coronary artery disease
kV: Kilo volt
UACR: Urine albumin to creatinine ratio
HU: Hounsfield unit
CP: Calcified plaque
SGLT2i: Sodium-glucose transport inhibitor
DPP4i: Dipeptidyl peptidase-4 inhibitor
IL-6: Interleukin-6
TNFα: Tumor necrosis factor-alpha
hs-CRP: High-sensitivity C-reactive protein
MACE: Major adverse cardiovascular events
GLP-1 RA: Glucagon-like peptide-1 receptor agonist
