# Supplementary material for: Pericoronary adipose tissue attenuation predicts compositional plaque changes: a 12-month longitudinal study in individuals with type 2 diabetes without symptoms or known coronary artery disease
Source: Cardiovasc Diabetol. 2025 Mar 28;24:143. doi: 10.1186/s12933-025-02694-9 (PMC11954229; doi:10.1186/s12933-025-02694-9)
Supplement: Supplementary file 1 — Supplementary Material 1 [file 12933_2025_2694_MOESM1_ESM.docx]

# Supplementary material 1

**Supplementary table 1**. Participant characteristics for the excluded participants

| **Demographic Data** | Included participants  *n* = 200 | Excluded participants  *n= 60* | *p-value* |
| --- | --- | --- | --- |
| Age, years | 61 ±9.4 | 62 ±11.5 | 0.8 |
| Sex, male | 153 (72) | 40 (67) | 0.8 |
| BMI, kg/m^2^ | 30.5 ±4.5 | 33.9 ±6.8 | 0.0001 |
| Waist-hip-ratio | 1.0 ±0.09 | 1.0 ±0.09 | 0.7 |
| Systolic BP, mmHg | 141 ±16 | 143 ±16 | 0.8 |
| Diastolic BP, mmHg | 86 ±10 | 8 6±11 | 0.6 |
| Smoking status: |  |  |  |
| Never smoker | 78 (39) | 24 (41) | 0.6 |
| Former smoker | 75 (38) | 24 (41) |  |
| Active smoker | 47 (24) | 10 (17) |  |
| Pack years | 9 (0-30) | 9 (0-25) | 1.0 |
| **Medications** |  |  |  |
| Antihypertensive medication | 137 (69) | 45 (75) | 0.3 |
| Statins | 150 (75) | 35 (58) | 0.01 |
| Metformin | 165 (83) | 46 (77) | 0.3 |
| Liraglutide | 51 (26) | 12 (20) | 0.4 |
| SGLT2i | 18 (9) | 7 (12) | 0.5 |
| Insulin | 77 (39) | 78 (45) | 0.4 |
| DPP4i | 30 (15) | 7 (12) | 0.7 |
| Sulfonylurea | 36 (18) | 8 (13) | 0.4 |
| **Diabetes-related** |  |  |  |
| Diabetes duration, year | 10 ±6 | 12 ±9 | 0.04 |
| Neuropathy | 47 (28) | 21 (40) | 0.2 |
| Retinopathy | 47 (24) | 11 (20) | 0.7 |
| Nephropathy | 56 (28) | 18 (30) | 0.6 |
| Number of DM complications |  |  |  |
| *0* | 98 (49) | 31 (52) | 0.7 |
| *1* | 66 (33) | 14 (23) | 0.2 |
| *> 2* | 35 (18) | 15 (25) | 0.2 |
| **Biochemistry** |  |  |  |
| HbA1c, mmol/mol | 60 ±14 | 63 ±17 | 0.2 |
| Total cholesterol, mmol/L | 4.1 ±1.0 | 4.1 ±1.2 | 1.0 |
| HDL, mmol/L | 1.1 (1-1.4) | 1.05 (0.9-1.5) | 0.5 |
| LDL, mmol/L | 2.0±0.8 | 2.0±1.0 | 0.6 |
| Triglycerides, mmol/L | 1.8 (1.3-2.5) | 1.9 (1.2-2.9) | 0.4 |
| eGFR, mL/min/1.73m^2^ | 90 (89-90) | 90 (89-90) | 0.6 |
| UACR, mg/g | 12 (6-37) | 15 (9-32) | 0.2 |
| CRP, mg/L | 1.5 (0.6-3.5) | 1.5 (0.6-4.6) | 0.3 |
| **CCTA** |  |  |  |
| CCS | 77 (1-475) | 113 (11-815) | 0.1 |

Values are mean ±standard deviation (SD), median + interquartile range (IQR), or counts (*n*) + proportions (*%*). BMI = body mass index; BP = blood pressure; SGLT2i = sodium-glucose cotransporter-2 inhibitor; DPP4i= dipeptidyl peptidase-4 inhibitor, DM = diabetes mellitus; HbA1c = glycated hemoglobin A1C; HDL = high-density lipoprotein; LDL = low-density lipoprotein; eGFR = estimated glomerular filtration rate; UACR = urinary albumin-to-creatinine ratio; CRP = C-reactive protein, CCTA= Coronary computed tomography angiography, CCS= Coronary calcium score.

**Supplemental Figure S1)** Correlation and scatterplot with fit-line between PCATa and CX, LAD and RCA PCATa, respectively.

**Supplementary Figure S2.**  Distribution of baseline PCATa

**Supplementary table 2.**  Participant characteristics for low vs. high PCATa

| **Demographic Data** | Low PCATa  *n* = 156 | High PCATa  *n= 44* | *p-value* |
| --- | --- | --- | --- |
| Age, years | 61 ±9.3 | 62 ±9.6 | 0.6 |
| Sex, male | 107 (69) | 38 (77) | 0.02 |
| BMI, kg/m^2^ | 31.1 ±4.6 | 28.3 ±3.8 | <0.001 |
| Waist-hip-ratio | 1.0 ±0.06 | 1.0 ±0.1 | 0.2 |
| Systolic BP, mmHg | 141 ±16 | 143 ±16 | 0.4 |
| Diastolic BP, mmHg | 87 ±10 | 84 ±11 | 0.1 |
| Smoking status: |  |  |  |
| Never smoker | 60 (38) | 18 (41) | 0.9 |
| Former smoker | 60 (38) | 15 (34) |  |
| Active smoker | 36 (23) | 11 (25) |  |
| Pack years | 10 (0-30) | 3 (0-21) | 0.4 |
| **Medications** |  |  |  |
| Antihypertensive medication | 112 (72) | 25 (56) | 0.06 |
| Statins | 120 (77) | 30 (68) | 0.2 |
| Metformin | 132 (85) | 33 (75) | 0.1 |
| Liraglutide | 44 (28) | 7 (16) | 0.1 |
| SGLT2i | 16 (10) | 2 (5) | 0.2 |
| Insulin | 59 (38) | 18 (41) | 0.4 |
| DPP4i | 21 (14) | 9 (20) | 0.3 |
| Sulfonylurea | 24 (15) | 12 (27) | 0.07 |
| **Diabetes-related** |  |  |  |
| Diabetes duration | 10 ±6 | 11 ±9 | 0.4 |
| Neuropathy | 35 (27) | 12 (27) | 0.2 |
| Retinopathy | 37 (25) | 10 (23) | 0.8 |
| Nephropathy | 42 (27) | 14 (31) | 0.6 |
| Number of DM complications |  |  |  |
| *0* | 77 (49) | 21 (48) | 0.8 |
| *1* | 49 (31) | 17 (39) | 0.4 |
| *> 2* | 29 (19) | 6 (14) | 0.5 |
| **Biochemistry** |  |  |  |
| HbA1c, mmol/mol | 60 ±14 | 61 ±17 | 0.5 |
| Total cholesterol, mmol/L | 4.1 ±1.0 | 4.1 ±1.2 | 0.9 |
| HDL, mmol/L | 1.1 (0.9-1.4) | 1.2 (1.1-1.5) | 0.03 |
| LDL, mmol/L | 2.0 ±0.8 | 2.1 ±1.0 | 0.8 |
| Triglycerides, mmol/L | 1.9 (1.4-2.7) | 1.4 (1.1-2) | 0.0007 |
| eGFR, mL/min/1.73m^2^ | 90 (88-90) | 90 (90-90) | 0.3 |
| UACR, mg/g | 12 (6-36) | 11 (6-39) | 0.5 |
| CRP, mg/L | 1.5 (0.6-3.5) | 1.2 (0.6-2.7) | 0.3 |
| **CCTA** |  |  |  |
| CCS | 75 (0-422) | 69 (1-632) | 1.0 |

Values are mean ±standard deviation (SD), median + interquartile range (IQR), or counts (*n*) + proportions (*%*). BMI = body mass index; BP = blood pressure; SGLT2i = sodium-glucose cotransporter-2 inhibitor; DPP4i= dipeptidyl peptidase-4 inhibitor, DM = diabetes mellitus; HbA1c = glycated hemoglobin A1C; HDL = high-density lipoprotein; LDL = low-density lipoprotein; eGFR = estimated glomerular filtration rate; UACR = urinary albumin-to-creatinine ratio; CRP = C-reactive protein, CCTA= Coronary computed tomography angiography, CCS= Coronary calcium score.

**Supplementary table 3.**  Compositional changes in plaque burden by low vs. high PCATa

| Δ **Plaque burden** | Low PCATa  *n* = 156 | | High PCATa  *n= 44* | |  |
| --- | --- | --- | --- | --- | --- |
|  | Means ±SD | 95% CI | Mean ±SD | 95% CI | p-value |
| Δ Total | 0.12 ±0.7 | 0.01-0.2 | 0.4 ±0.6 | 0.2-0.5 | 0.04 |
| Δ Calcified | 0.07 ±0.2 | 0.04-0.1 | 0.09 ±0.2 | 0.04-0.2 | 0.6 |
| Δ Non-calcified | 0.04 ±0.6 | -0.04-0.1 | 0.3 ±0.5 | 0.09—0.4 | 0.03 |

Plaque burden changes followed a normal distribution. Means ± standard deviation (SD) or medians (IQR) are presented. Unpaired t-test was used. Δ represents changes over 12 months. CI= Confidence Interval.
